# Oyster broth concentrate and its major component taurine alleviate acute alcohol‐induced liver damage

**DOI:** 10.1002/fsn3.2847

**Published:** 2022-03-29

**Authors:** Adrian S. Siregar, Marie Merci Nyiramana, Eun‐Jin Kim, Eui‐Jung Shin, Min Seok Woo, Jin‐Mok Kim, Si‐Hyang Park, Jong Ryeal Hahm, Yeung Joon Choi, Dawon Kang

**Affiliations:** ^1^ Department of Physiology and Institute of Health Sciences College of Medicine Gyeongsang National University Jinju South Korea; ^2^ Department of Convergence Medical Science Gyeongsang National University Jinju South Korea; ^3^ Department of Clinical Laboratory Science Masan University Changwon South Korea; ^4^ Sunmarin Biotech Jinju Bioindustry Foundation Jinju South Korea; ^5^ Department of Internal Medicine, Hospital and Institute of Health Sciences College of Medicine Gyeongsang National University Jinju South Korea; ^6^ Ocean‐Pep Jinju Bioindustry Foundation Jinju South Korea

**Keywords:** alcohols, functional foods, inflammation, liver injury, oxidative stress, oyster broth concentrate

## Abstract

Our previous study showed that oyster hydrolysate (OH) protected against the liver damage caused by a single instance of ethanol (EtOH) binge drinking. Oyster broth concentrate (OBC) was discovered in the process of searching for a different substance derived from oysters (*Crassostrea gigas*) with economic value. OBC is a by‐product of boiling oysters at 95°C for 3 min. In this study, we investigated the effects of OBC and its major component taurine on blood and liver tissues obtained from a single‐EtOH‐binge‐drinking mouse model. The preadministration of OBC enhanced EtOH metabolism by increasing the activities of alcohol dehydrogenase (ADH), aldehyde dehydrogenase (ALDH), and catalase. In addition, the preadministration of OBC reduced cytochrome P450 2E1 (CYP2E1) activity, reactive oxygen species (ROS) generation, Ca^2+^ concentrations, apoptotic signals, and inflammatory mediators in liver tissues. The reduction of apoptotic and inflammatory signals by OBC resulted from the downregulation of endoplasmic reticulum (ER) stress molecules and NF‐κB activity. Taurine administration showed similar effects to OBC. These results show that OBC protected against acute EtOH‐induced liver damage through the action of taurine. Our findings suggest that OBC could be an economically valuable substance and a functional food with hepatoprotective effects.

## Graphical abstract

OBC is a functional food that can be made very simply by boiling oysters at 95°C for 3 min. Taurine is a major component of OBC, accounting for 41.8% of the total free amino acids analyzed. Preadministration of OBC or taurine enhanced EtOH metabolism and reduced ER stress, apoptotic signals, and inflammatory mediators in a single‐EtOH‐binge mouse model.

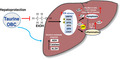

## 1 INTRODUCTION

In modern society, alcohol consumption is increasing and becoming part of culture. Excessive alcohol consumption is the most common form of drug abuse among modern people and causes serious health problems, especially liver and brain damage. Frequent alcohol intake is known to affect hepatocellular disorders and metabolic processes in the body. However, our recent study has shown that excessive alcohol consumption, regardless of the frequency, causes liver toxicity and damage (Siregar et al., 2020). Approximately 90% of the alcohol we drink is absorbed in the liver. Alcohol is oxidized to acetaldehyde by the action of alcohol dehydrogenase (ADH), catalase, and cytochrome P450 2E1 (CYP2E1). Acetaldehyde is then converted to acetate by aldehyde dehydrogenase (ALDH), a process that occurs primarily in the liver (Jiang et al., 2020).

Hepatocytes are rich in endoplasmic reticulum (ER). The unfolded protein response (UPR) plays a pivotal role in maintaining ER homeostasis in the liver under physiological and pathological conditions. Alcohol‐derived acetaldehyde, a major cause of liver damage, causes ER stress and injury (Ji, 2012). Alcohol alters the expression of glucose regulatory protein 78 (GRP78) and sterol regulatory element‐binding proteins (SREBPs), which are associated with ER stress (Malhi & Kaufman, 2011). GRP78 interacts with three ER transmembrane sensors (inositol‐requiring enzyme‐1 (IRE1)α, transcription factor‐6 (ATF6), and protein kinase R‐like endoplasmic reticulum kinase (PERK)) via the PERK/eukaryotic initiation factor 2α (eIF‑2α) and IRE1α or ATF6/X‐box binding protein (XBP)‑1 axes, which are two branches of the UPR, affecting the expression of CCAAT‐enhancer‐binding protein homologous protein (CHOP) (Adams et al., 2019; Kouznetsova et al., 2018). CHOP is a key factor in cell death induced by the alcohol‐induced ER stress (Malhi & Kaufman, 2011). Alcohol‐induced ER stress has emerged as an alternative mechanism behind alcohol hangovers and liver damage. ER stress is closely related to apoptosis and inflammation (Koo & Han, 2021; Liu & Green, 2019). However, there are few studies of the interaction between a single instance of excessive alcohol intake and the progression of ER stress in the liver.

Our previous study reported that oyster (*Crassostrea gigas*) hydrolysate (OH) enhanced ethanol (EtOH) metabolism and had hepatoprotective effects in a single‐EtOH‐binge rodent model (Siregar et al., 2020). Its effects are attributed to the tyrosine alanine (YA) peptide, which is the main component of OH. OH contains more amino acids and low‐molecular‐weight peptides than oyster extract because it is produced by enzymatic hydrolysis. Besides OH, there are oyster products that are easy to make. An example is oyster broth concentrate (OBC), which is produced by boiling oysters at 95°C for 3 min. While the oysters are boiling, the oysters secrete their contents into the water, and their self‐cooking solution is concentrated to the expected brix. OBC is rich in glycogen and free amino acids. OBC has a higher mineral content than raw oysters (Ryu et al., 2015) and a higher content of free amino acids, such as taurine, proline, glycine, alanine, and β‐alanine than fermented oyster solution (Jeong et al., 2017a). It has been found that about 80% of the total amino acid content of oysters is taurine (Hosoi et al., 2003; Jeong et al., 2017b). Little is known about the effect of OBC on EtOH‐induced liver injury.

Taurine, a β‐amino sulfonic acid, is a major intracellular amino acid that shows various physiological functions in the human body. Numerous studies have demonstrated that taurine plays an important role in hepatoprotection in vitro and in vivo (Song et al., 2021; Uzunhisarcikli & Aslanturk, 2019). Changes in hepatic taurine levels have been suggested as an indicator of hepatic dysfunction and biochemical disturbances (Timbrell & Waterfield, 1996). Taurine abates the liver damage induced by γ‐irradiation in rats through anti‐inflammatory and anti‐apoptotic pathways (El‐Maraghi et al., 2020). In addition, dietary taurine helps to reduce the liver inflammation and ER stress induced by high dietary carbohydrate intake (Zhang et al., 2021). Taurine administration also decreases the activity of aspartate transaminase (AST) and alanine transaminase (ALT), and increases that of ADH and ALDH in chronic alcoholic patients (Hsieh et al., 2014). Taurine shows beneficial effects on liver disease caused by chronic alcohol consumption in human and animal models (Hsieh et al., 2014; Song et al., 2021). This study was performed to determine whether OBC rich in taurine had a hepatoprotective effect in a single‐EtOH‐binge mouse model.

## 2 MATERIALS AND METHODS

### 2.1 Oyster broth concentrate and oyster hydrolysate

Oyster broth concentrate (OBC), a by‐product produced when oysters are boiled at 95°C for 3 min, was purchased from an oyster‐canning processing plant in Tongyeong (Korea). OBC showing brix 45.00 ± 0.16% was used in this study. The oyster hydrolysate was prepared according to a previous protocol (Siregar et al., 2020).

### 2.2 Measurement of radical scavenging activity and inflammatory enzyme inhibiting activity of OBC

The 2.2‐diphenyl‐1‐picrylhydrazyl (DPPH) and 2,2‐azinobis‐(3‐ethylbenzothiazoline‐6‐sulfonate) (ABTS) radical scavenging activities of OBC were measured according to a previous protocol (Siregar et al., 2020). The percentages of cyclooxygenase‐2 (COX‐2) inhibition and 5‐lipoxygenase (5‐LO) inhibition were measured according to a previous protocol (Siregar et al., 2020).

### 2.3 Alcohol intoxication model

The animal experiments were performed in accordance with the guidelines of the Gyeongsang National University animal care and use committee (GNU‐201012‐M0073). Male C57BL/6 mice (7 weeks old) were purchased from Koatech Co. (Animal Breeding Center, Pyeongtaek, Korea). The mice were maintained under a 12‐h light/dark cycle in a specific pathogen‐free animal facility with food and water freely available for 1 week prior to the experiment. All the mice were randomly separated into the following five groups: vehicle (saline + saline), EtOH (3 g/kg) + saline, EtOH + OBC (200 mg/kg), EtOH + OH (200 mg/kg), and EtOH + taurine (45 mg/kg). EtOH was administered, and saline, OH, OBC, and taurine were preadministered 1 h before EtOH administration by oral gavage. Blood was drawn from the heart 7 h after EtOH administration. Liver tissues were quickly isolated and placed into a deep freezer at −80°C or a 4% paraformaldehyde solution for further experiments.

### 2.4 Rotarod performance test

Motor coordination and balance were evaluated using a rotarod apparatus (B1001‐006, B.S Technolab Inc.). The apparatus consisted of five lanes with a 30 mm rotor diameter, 50 mm lane width, 200 mm falling height, and 250 mm lane separator diameter. All the mice were evaluated on a rotarod according to a previous protocol (Siregar et al., 2020).

### 2.5 Measurement of Ethanol (EtOH) concentration

The plasma EtOH concentration was measured using an Ethanol Assay Kit (Abcam) according to the manufacturer's protocol, as previously described (Siregar et al., 2020).

### 2.6 Detection of acetaldehyde concentration

The concentration of acetaldehyde in the plasma was measured using an EnzyChrom™ Acetaldehyde Assay Kit according to the manufacturer's protocol (BioAssay Systems), as previously described (Siregar et al., 2020).

### 2.7 Measurement of Alcohol Dehydrogenase (ADH) and NAD‐Dependent Aldehyde Dehydrogenase (ALDH) activity

The activity of ADH and ALDH in the plasma was measured using an Alcohol Dehydrogenase Assay Kit (Abcam) and an ALDH Activity Assay Kit, respectively, according to the manufacturer's protocol, as previously mentioned (Siregar et al., 2020).

### 2.8 Measurement of catalase and CYP2E1 activity

Mouse liver tissues were homogenized in 50 mM phosphate buffer (pH 7.4) containing 1% Triton X‐100 and in 5 ml of 0.15 M KCl, respectively, for measuring the catalase and CYP2E1 activity. The activity was then measured according to a previous protocol (Siregar et al., 2020).

### 2.9 Measurement of Alanine Aminotransferase (ALT) and Aspartate Aminotransferase (AST) levels

The ALT and AST levels in the serum were measured by GC Labs (Yongin, Korea), which used the International Federation of Clinical Chemistry standard method, according to a previous protocol (Siregar et al., 2020).

### 2.10 Measurement of total free radical activity in tissues

The total free radical activity in liver tissue lysates was measured using the Oxiselect™ In Vitro ROS/RNS assay kit (Cell Biolabs) according to the manufacturer's protocol, as previously mentioned (Siregar et al., 2020).

### 2.11 Measurement of calcium concentration in tissues

The calcium concentration was measured using a Calcium Detection Assay Kit (Abcam) according to the manufacturer's protocol. Liver tissues (100 mg) were homogenized in 500 µl of calcium assay buffer and then sonicated for 10 s in an ice bath with a VCX‐500 Ultrasonic Processor (Sonics and Materials Inc.). The homogenate was centrifuged at 12000 × *g* for 5 min (Eppendorf Centrifuge 5424R, Eppendorf AG). After centrifugation, the supernatant was transferred to a clean tube and used as the tissue lysate. Calcium standards were prepared in serial dilutions of 0, 0.2, 0.4, 0.6, 0.8, and 1 mM by diluting the 5 mM standard stock in distilled water. The reaction was set up by mixing 50 µl of calcium standard or tissue lysates, 90 µl of chromogenic reagent, and 60 µl of calcium assay buffer into a 96‐well plate, and then incubating for 10 min at room temperature in the dark. The absorbance at 575 nm was measured using a VERSAmax™ microplate reader (Molecular Devices). The calcium concentration (mM) was calculated by dividing the sample amount from the standard curve by the sample volume added into the well and multiplying by the sample dilution factor.

### 2.12 Hematoxylin and Eosin (H&E) staining

Histological changes in the liver tissue were analyzed by H&E staining (Sigma Aldrich). Mice were perfused with a fixative solution containing 4% paraformaldehyde solution, and liver was isolated and incubated in the same fixative solution overnight at 4°C. The liver tissues were embedded in paraffin after washing three times. The paraffin blocks were sectioned to a thickness of 5 mm and air‐dried on gelatin‐coated slides. For H&E staining, the paraffin was removed from the liver tissue sections with xylene, and the sections were rehydrated with graded alcohol series (100%–70% EtOH). The liver tissue section was washed with tap water for 5 min, and the section slide was immersed in hematoxylin solution for 5 min. After checking the degree of hematoxylin staining, eosin staining was performed for 1 min. The sections were dehydrated through a graded series of EtOH (70%–100% EtOH, each 3 min), removed from xylene, and mounted with mounting medium (Fisher Chemical). The stained part was photographed using a BX61VS microscope (Olympus).

### 2.13 Immunohistochemistry

The deparaffinized tissue sections were washed in PBS and permeabilized with 0.2% Triton X‐100 for 10 min at room temperature. After washing three times in PBS, the sections were incubated for 1 h at room temperature in a blocking buffer containing 1% normal goat serum in PBS, followed by incubation with anti‐CD68 antibody (1:200 dilution, Santa Cruz Biotechnology) overnight at 4°C. Then, they were rinsed three times in PBS and incubated for 1.5 h in the dark with Alexa Fluor 488‐conjugated goat anti‐rabbit IgG diluted 1:400 in PBS (Thermo Scientific Fisher/Invitrogen). Finally, tissue sections were washed three times in PBS and stained with Hoechst 3386 (1.0 mg/ml, Sigma Aldrich) for 10 min at room temperature. After washing three times, the tissue section was mounted with Permount Mounting Medium (Fisher Chemical). Images were captured using the IX70 Fluoview confocal laser scanning microscope equipped with a fluorescence system (Olympus).

### 2.14 Immunoassay for IL‐1β, IL‐6, and TNF‐α cytokines in tissues

The concentrations of the pro‐inflammatory cytokines IL‐1β, IL‐6, and TNF‐α in the liver tissues were quantified using an ELISA kit (R&D system, Minneapolis, MN, USA) according to the manufacturer's protocol. Liver tissues (50 mg) homogenized in 1 ml of 1 × PBS were sonicated for 10 s in an ice bath with a VCX‐500 Ultrasonic Processor (Sonics and Materials Inc.). The homogenates were subjected to centrifugation at 12,000 × *g* for 5 min (Eppendorf Centrifuge 5424R, Eppendorf AG). After centrifugation, the supernatant was transferred to a clean tube and used as the tissue lysate. Tissue lysates (50 μl) were added to the wells of 96‐well plates, which were precoated with anti‐IL‐1β, IL‐6, and TNF‐α antibodies. The plates were covered with an adhesive strip, incubated for 2 h at room temperature, and washed three times with a wash buffer. Then, 100 μl of mouse IL‐1β, IL‐6, and TNF‐α conjugate was added, and the plates were incubated for 2 h at room temperature, and washed three times. The reaction was quenched by the addition of 100 μl of stop solution. The absorbance of the plates at 450 nm was read with a microplate reader (Molecular Devices).

### 2.15 RT‐PCR

Total RNA isolated from mouse liver tissues was used to synthesize first‐strand cDNA using a reverse transcriptase kit (DiaStartTM RT kit; SolGent). The cDNA, mouse‐specific gene primers, and Taq polymerase (G‐Taq, Cosmo Genetech) were used for PCR. The sequences of the primers are listed in Table 1. Glyceraldehyde‐3‐phosphate dehydrogenase (GAPDH) was used as a loading control. The PCR conditions included an initial denaturation at 94°C for 5 min, followed by 30 cycles of 94°C for 30 s, 58°C for 30 s, and 72°C for 30 s, and a final extension step at 72°C for 10 min. The PCR product size was confirmed by electrophoresis on a 1.5% (w/v) agarose gel, and the PCR products were directly sequenced with the ABI PRISM^®^ 3100‐Avant Genetic Analyzer (Applied Biosystems). Images of the DNA fragments were captured with the iBright™ CL1500 Imaging System (Thermo Scientific Fisher/Life Technologies Holdings Pte Ltd.).

**TABLE 1:** Primer sequences used for PCR

Gene Name	GenBank Accession No.	Primer Sequences (5′‐3′)		Expected Size (bp)
IL‐1β	NM_008361.4	F	TGAAGAAGAGCCCATCCTCTG	440
		R	CTTGTGAGGTGCTGATGTACC	
IL‐6	NM_031168	F	CTTCACAAGTCCGGAGAGGAG	489
		R	TGGTCTTGGTCCTTAGCCACT	
TNF‐α	NM_013693.3	F	CAGCCTCTTCTCATTCCTGCT	339
		R	TGTCCCTTGAAGAGAACCTGG	
GAPDH	NM_017008	F	CTAAAGGGCATCCTGGGC	201
		R	TTACTCCTTGGAGGCCATG	

### 2.16 Western Blot analysis

Total protein was isolated from liver tissue using the RIPA buffer (Thermo Fisher Scientific) containing 1× protease inhibitor cocktail (Roche Diagnostics). Liver tissues homogenized in a hypotonic lysis buffer were centrifuged at 142 *g* for 5 min at 4°C to isolate nuclear and cytosolic fractions. The resulting pellets and supernatants were processed for the isolation of the nuclear and cytosolic fractions, respectively. The pellets were incubated on ice for 5 min in a nuclear isolation buffer and were spun at 142 g (Eppendorf Centrifuge 5424R, Eppendorf AG) at 4°C for 5 min. The resulting pellets were incubated on ice for 30 min with 2% Triton nuclear isolation buffer and then spun at 12,448 g at 4°C for 15 min. For the cytosolic fraction, the supernatants in the hypotonic lysis buffer were transferred to a new 1.5 ml tube and spun at 15,401 g for 15 min at 4°C. The resulting supernatants were centrifuged at 100,000 *g* at 4°C for 60 min in an ultracentrifuge (TLA 100.3; Optima MAX‐XP, Beckman Coulter, Inc.). The procedures for the western blot analysis and mitochondrial and cytosolic fractionation were performed as previously described (Siregar et al., 2020). The membranes were blocked with 5% (w/v) fat‐free dry milk in TBS with Tween‐20 (TBST; 20 mM Tris HCl (pH 8.0), 137 mM NaCl, and 0.2% Tween‐20) at room temperature for 60 min and then incubated with anti‐Bax (1:200 dilution; Santa Cruz Biotechnology), anti‐Bcl‐2 (1:200 dilution; Santa Cruz Biotechnology), anti‐cytochrome C (1:1,000; Cell Signaling), anti‐VDAC (1:1000; Cell Signaling), anti‐caspase‐3 (1:1000; Cell Signaling), anti‐NF‐κB (1:1000; Cell Signaling), anti‐GRP78 (1:1000 dilution, Abcam), anti‐pERK (1:200 dilution; Santa Cruz Biotechnology), anti‐p‐pERK (1:200 dilution; Santa Cruz Biotechnology), anti‐elF2α (1:1000; Cell Signaling), anti‐p‐elF2α (1:1000; Cell Signaling), anti‐ATF4 (1:200 dilution; Santa Cruz Biotechnology), anti‐ATF6 (1:1000; Cell Signaling), anti‐CHOP (1:200 dilution; Santa Cruz Biotechnology), anti‐Lamin (1:200 dilution; Santa Cruz Biotechnology), and anti‐β‐actin antibodies (1:5000 dilution; Thermo Scientific Fisher/Invitrogen) at 4°C overnight. The primary antibody incubation was followed by incubation with a secondary HRP‐conjugated anti‐rabbit or anti‐mouse antibody at 1:10,000 (Assay Designs). Immuno‐positive bands were enhanced with the chemiluminescence Super Signal™ West Pico PLUS Luminol/Enhancer (Thermo Fisher Scientific/Pierce Biotechnology) and visualized using the iBright™ CL1500 imaging system (Thermo Scientific Fisher/Life Technologies Holdings Pte Ltd.).

### 2.17 Data analysis and statistics

The bands in the images of the western blots and agarose gels captured using an iBright™ CL1500 imaging system (Thermo Scientific Fisher/Life Technologies Holdings Pte Ltd.) were quantified using ImageJ (version 1.49, National Institutes of Health, Bethesda, MD, USA). The data are presented as mean ± standard deviation (SD). A one‐way ANOVA/Bonferroni test or the Kruskal–Wallis/Mann–Whitney test was chosen after the normality test to analyze the differences among groups (OriginPro2020, OriginLab Corp.). A *p* < .05 was considered as the criterion for statistical significance.

## 3 RESULTS

### 3.1 Effect of oyster broth concentrate on liver damage in single‐binge EtOH‐fed mice

**FIGURE 1:**
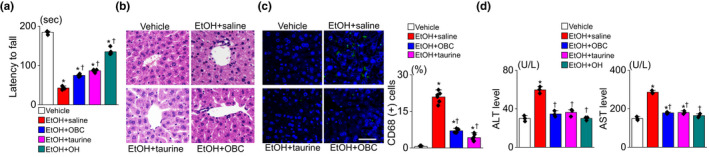
Reduction of EtOH‐induced hepatic damage by OBC preadministration. All experiments were performed using samples (liver and blood) obtained at 7 h after EtOH administration. OBC, taurine, and OH were orally preadministered 1 h before EtOH administration. (a) Rotarod test. Latency to fall was measured at 7 h after EtOH administration. (b and c) Histological analysis. Hematoxylin and eosin staining (B) and CD68 immunostaining (c). Hoechst staining (blue) was performed for nuclear staining (×400) in anti‐CD68 immunostaining. Scale bar represents 40 µm. (D) ALT and AST levels in blood. Each bar represents means ± SD of three independent experiments. *p < .05 compared to vehicle group; ^†^ *p* < .05 compared to EtOH + saline group

OBC showed antioxidant and anti‐inflammatory activity as judged by measuring the radical scavenging activity and cyclooxygenase‐2 (COX‐2) and 5‐lipoxygenase (5‐LO) activity (Figure 1). As shown in Table S1, the taurine content accounted for 41.8% of the total free amino acids in the OBC used in this study. The concentration of taurine in the OBC was four times higher than that in OH. Motor coordination and balance were assessed in a Rotarod performance test 7 h after EtOH administration. The group preadministered OBC (200 mg/kg), OH (200 mg/kg), or taurine (45 mg/kg) prior to EtOH (3 g/kg) administration remained for significantly longer on the rotating rod than the group administered EtOH alone (EtOH + saline group) (each group: *n* = 15, *p* < .05, Figure 1a). Liver tissues obtained from each group were adopted for hematoxylin and eosin (H&E) staining and immunohistochemistry. The H&E staining showed hepatocellular damage, with a contracted cytoplasm and nucleus and numerous lipid vacuoles visible in the cytoplasm of the hepatocytes in the liver tissue obtained from the EtOH + saline group, and the hepatocellular damage was prevented in the EtOH + OBC and EtOH + taurine groups (Figure 1b). The immunofluorescence intensity for CD68, a marker of macrophages, was higher in the EtOH + saline group, indicating that EtOH induces liver inflammation. The number of macrophages showing CD68‐positive green signals was significantly decreased in both the EtOH + OBC and EtOH + taurine groups (*n* = 5, *p* < .05, Figure 1c). The serum levels of AST and ALT were higher in the EtOH + saline group than in the vehicle group, while the ALT and AST levels were significantly decreased in the EtOH + OBC, EtOH + taurine, and EtOH + OH groups (*n* = 15, *p* < .05, Figure 1d). The EtOH + OH group was used as a positive control and showed an increase in EtOH metabolism and decrease in liver damage (Siregar et al., 2020).

### 3.2 OBC‐induced enhancement of alcohol metabolism

**FIGURE 2:**
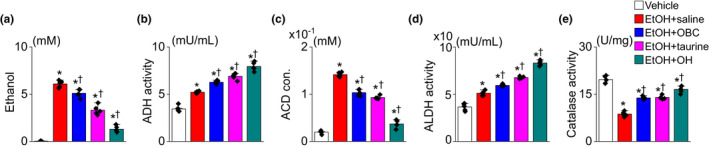
Increase in hepatic activity of EtOH metabolizing enzymes with OBC preadministration. The samples were obtained 7 h after EtOH administration. (a) Blood EtOH concentration. (b) Blood alcohol dehydrogenase activity. (c) Blood acetaldehyde concentration. (d) Blood aldehyde dehydrogenase activity. (e) Catalase activity in liver tissue lysate. Each bar graph represents means ± SD of three independent experiments. *p < .05 compared to vehicle group; ^†^ *p* < .05 compared to EtOH + saline group

The EtOH concentrations were measured at 7 h after EtOH administration. The blood EtOH concentration was significantly decreased in the EtOH + OBC, EtOH + taurine, and EtOH + OH groups compared to the EtOH + saline group (*p* < .05; each group: *n* = 12, Figure 2a). The ADH activity increased in each group as the EtOH concentration decreased, in the following order: EtOH + OBC, EtOH + taurine, and EtOH + OH (*p* < .05; each group: *n* = 12, Figure 2b). The concentration of acetaldehyde and the activity of ALDH and catalase were changed similar to the EtOH concentration and ADH activity in each group (*p* < .05, *n* = 12, Figure 2c to e).

### 3.3 Protective effect of OBC on ER stress‐induced hepatic apoptosis

**FIGURE 3:**
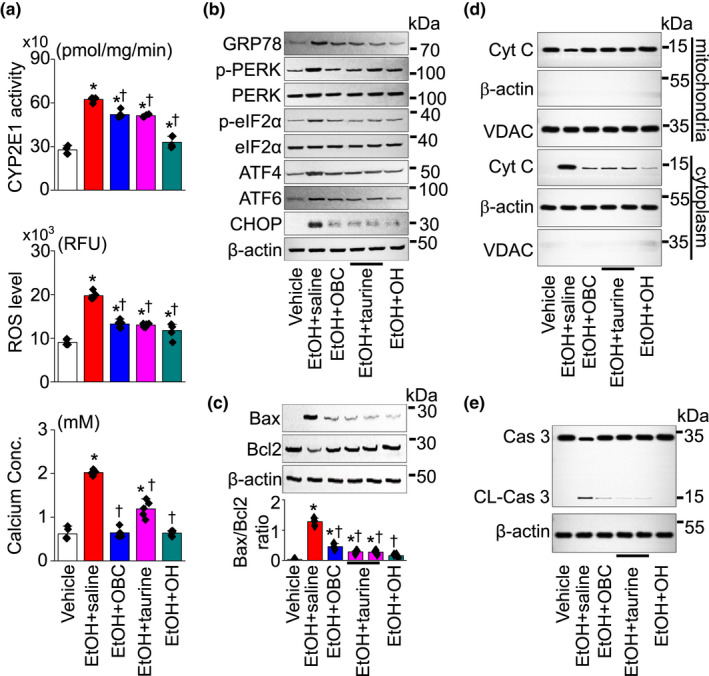
Reduction of EtOH‐induced apoptotic signals by OBC. (a) CYP2E1 activity, ROS/RNS free radical activity, and Ca^2+^ concentration measured in liver tissue lysates. (b) Western blot analysis of ER stress molecules. (c) Analysis of proapoptotic Bax and anti‐apoptotic Bcl‐2 ratio. (d) Cytochrome C (Cyt C) release from mitochondria to cytoplasm. Liver tissue lysates were fractionated into mitochondria and cytoplasm components. Mitochondrial fraction was confirmed by expression of voltage‐dependent anion channel (VDAC), which is expressed in the outer mitochondrial membrane, and no expression of β‐actin. (e) Analysis of caspase 3 cleavage. An aliquot of cell lysate (30 μg of protein per lane) was analyzed by immunoblotting. Each bar represents mean ± SD of three independent experiments. *p <.05 compared to vehicle; ^†^ *p* < .05 compared to EtOH + saline group

The activity of CYP2E1, an active CYP450 isoform involved in generating intracellular reactive oxygen species (ROS), was high in the EtOH + saline group, while the activity was significantly decreased in the EtOH + OBC, EtOH + taurine, and EtOH + OH groups (*p* <.05; each group: *n* = 5, Figure 3a). The ROS generation and Ca^2+^ levels in the liver tissue lysates were also significantly increased in EtOH + saline group compared to the vehicle group, while their levels were significantly decreased in the EtOH + OBC, EtOH + taurine, and EtOH + OH groups compared to the EtOH + saline group (*p* < .05; each group: *n* = 5, Figure 3a). An increase in ROS generation and Ca^2+^ concentration is linked to ER stress. The ER stress markers GRP78, p‐PERK, p‐eIF2α, ATF4, ATF6, and CHOP were detected in the liver tissues. All the markers tested were upregulated in the EtOH + saline group, and the EtOH‐induced upregulation was decreased in EtOH + OBC, EtOH + taurine, and EtOH + OH groups (Figure 3b). Apoptotic markers (Bax/Bcl2 ratio, cytochrome C release, and caspase 3 cleavage) were also detected in the liver tissues. In the EtOH + saline group, the pro‐apoptotic Bax protein increased, whereas the anti‐apoptotic Bcl‐2 protein decreased. The Bax/Bcl‐2 ratio increased significantly in the EtOH + saline group, but the ratio was significantly decreased in the EtOH + OBC, EtOH + taurine, and EtOH + OH groups (*n* = 3, *p* < .05; Figure 3c). Cytochrome (Cyt) C release from mitochondria to the cytoplasm and caspase 3 cleavage were increased in the EtOH + saline group, but they decreased in the EtOH + OBC, EtOH + taurine, and EtOH + OH groups (*n* = 3, Figure 3d and e).

### 3.4 Anti‐inflammatory effect of OBC on EtOH‐induced hepatic inflammation

**FIGURE 4:**
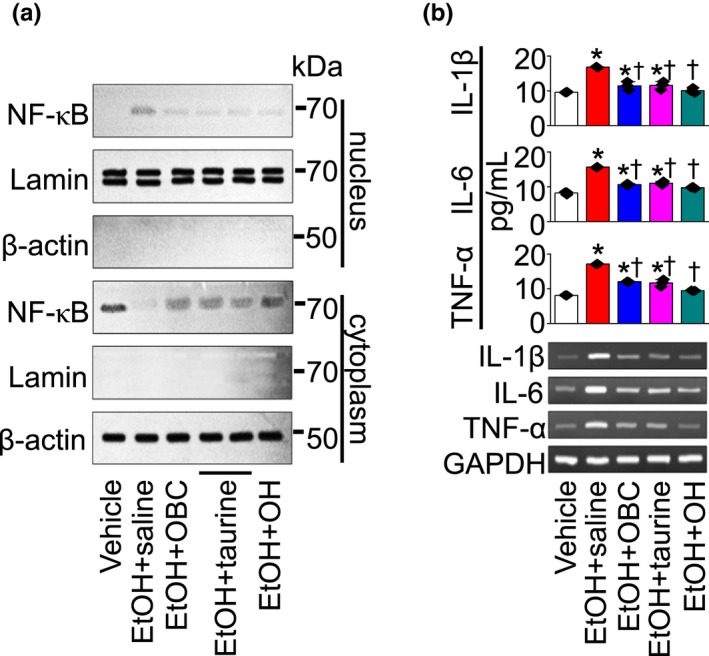
Reduction of EtOH‐induced inflammatory signals by OBC. Liver tissues were obtained from mice 7 h after EtOH administration. (a) NF‐κB translocation. Liver tissue lysates were fractionated into nucleus and cytoplasm components. Nucleus fraction was confirmed by expression of lamin, a nuclear structural protein, and no expression of β‐actin. An aliquot of cell lysate (30 μg of protein per lane) was analyzed by immunoblotting. (b) Analysis of pro‐inflammatory cytokine (IL‐1β, IL‐6, and TNF‐α) concentrations and expression by ELISA and semi‐quantitative PCR, respectively. Each bar represents mean ± SD of three independent experiments. *p < .05 compared to vehicle; ^†^ *p* < .05 compared to EtOH + saline group

The activation of nuclear factor kappa B (NF‐κB) was evaluated in EtOH‐fed liver tissue. Nuclear NF‐κB translocation was observed in the EtOH + saline group, while the translocation was inhibited in the EtOH + OBC, EtOH + taurine, and EtOH + OH groups (*n* = 3, Figure 4a). The concentrations and expression of IL‐1β, IL‐6, and TNF‐α were high in the EtOH + saline group compared to the vehicle group, whereas the EtOH‐induced increase in inflammatory mediators was significantly decreased in the EtOH + OBC, EtOH + taurine, and EtOH + OH groups compared to the EtOH + saline group (*p* < .05; *n* = 6, Figure 4b).

All the parameters analyzed in this study were also investigated in the group administered taurine alone. There were no significant differences between the groups vehicle and taurine alone, indicating that taurine has a significant effect in the context of EtOH metabolism rather than under normal conditions (Table S2).

## 4 DISCUSSION

This study explored alterations in liver factors 7 h after EtOH administration. Our previous study demonstrated the hepatoprotective effect of OH and YA peptide in a single‐EtOH‐binge model 5 h after alcohol administration (Siregar et al., 2020). At 5 h after EtOH exposure, the AST levels did not change. Other studies have recommended assessing ALT and AST levels 6 h after liver injury (Chen et al., 2016; Wang, Zhang, et al., 2016). Single‐EtOH‐binge administration increased hepatocellular degeneration, lipid deposition, and macrophage infiltration in liver tissue, and the ALT and AST levels in the blood 7 h after administration. The previous 5 h EtOH samples showed no significant histological changes in the liver (Siregar et al., 2020). These results indicate that molecular changes proceed morphological and histological changes.

The early detection of molecular markers is important in assessing liver damage. Many molecules are changed by the acetaldehyde produced after the administration of EtOH. In addition to acetaldehyde, changes in the redox state during alcohol removal can lead to liver damage (Jiang et al., 2020). EtOH metabolism is achieved in the liver by the collaboration of ADH, catalase, CYP2E1, and ALDH (Jiang et al., 2020). EtOH metabolism increases ROS production, which activates mitochondrial apoptosis pathways (Wang et al., 2016) and inflammatory mediators (Zheng et al., 2019). ER stress molecules are also related to EtOH‐induced oxidative and inflammatory damage. EtOH increases the accumulation of various misfolded proteins and subsequently promotes ER stress (Shinohara et al., 2010). In this study, a single binge EtOH administration upregulated CYP2E1 activity, ROS generation, the Ca^2+^ concentration, apoptotic and inflammatory signals, and ER stress molecules including CHOP. CHOP is a key factor in the cell death induced by EtOH‐induced ER stress (Ji et al., 2005). Substances that properly regulate or reduce these molecules need to be developed to effectively prevent and treat alcohol‐induced liver damage.

Oysters are a nutritionally balanced health food, such that they are referred to as sea milk. They are easy to incorporate into the diet compared to other natural products with anti‐hangover and hepatoprotective activity, such as pueraria lobata, fructus evodiae, trigonella foenum‐graecum, and hovenia dulcis (Kim et al., 2017; Wang, Li, et al., 2016). Oysters contain large amounts of protein, glycogen, taurine, iron, zinc, and vitamin C, and these ingredients are known to reduce oxidative and inflammatory stress, relieve hangovers, and improve blood circulation (Kim et al., 2015; Wang et al., 2010). Various processing methods have been developed to improve these nutrients and their biological activity, which can increase their effectiveness as functional foods. Increasing its economic value in addition to nutrients and biological activity would make it a better functional food with the potential to be developed as a pharmaceutical.

OBC is a product in which the functional ingredients of oysters are increased without using any enzymes or substances. In particular, the taurine concentration is very high in OBC. OBC is mainly used as a basic solution for making oyster sauce (Jeong et al., 2017a), and little research has been conducted on its physiological functions. This study was conducted to confirm the effect of OBC on liver damage under the hypothesis that OBC could protect against EtOH‐induced liver damage, because taurine, which is abundant in OBC, has already shown the ability to reduce liver damage. If OBC has bioactivity, it could be an excellent and economical functional food. We found that OBC has hepatoprotective effects in a single‐EtOH‐binge mouse model. The OBC used in this study had higher economic value than other compounds, such as OH and oyster extracts. The manufacture of OH requires an enzymatic procedure, and oyster extracts require a long procedure. OBC, on the other hand, is a concentrated soup obtained by simply boiling oysters at 94°C for 3 min. OBC promoted EtOH metabolism and had antioxidant, anti‐inflammatory, and anti‐apoptotic effects. The preadministration of taurine showed a hepatoprotective effect similar to that of OBC. The hepatoprotective effect of OBC was less than that of OH. However, considering the economics, OBC is as valuable as OH as a functional food.

Taurine is an endogenous metabolite distributed in many tissues at high concentrations. The liver is the main organ capable of synthesizing taurine. Taurine deficiency in cells and tissues causes serious pathological changes. Taurine protects cells by regulating oxidation, energy metabolism, gene expression, ER stress, neural activity, Ca^2+^ concentrations, and osmolality, thus protecting against neurodegenerative, cardiovascular, metabolic, inflammatory, and muscular diseases (Schaffer & Kim, 2018). Taurine is synthesized endogenously from cysteine but is mainly introduced by a seafood‐rich diet (Bouckenooghe et al., 2006). A semi‐essential beta amino acid taurine shows protective effects on the liver in vitro and in vivo. In alcoholic fatty liver disease, taurine significantly inhibits liver damage through the stimulation of alcohol metabolism by ADH and ALDH (*p* < .05), as well as the metabolism of fat (Wu et al., 2015). Taurine prevents EtOH‐induced apoptosis mediated by mitochondrial or death‐receptor pathways in liver cells (Wu et al., 2018). Consistent with earlier studies, our findings also show that taurine protects against EtOH‐induced liver damage.

In addition to taurine, glycine, proline, alanine, arginine, and glutamate are abundant in OBC (Table S1). These amino acids regulate various aspects of cellular metabolism and detoxification in the liver (Lee & Kim, 2019) through their antioxidant and anti‐inflammatory properties (Heidari et al., 2018; Maezono et al., 1996; Nanji et al., 2001; Senthilkumar et al., 2004) (Wu et al., 2004). The action of these amino acids is presumed to have contributed to the hepatoprotective effect of OBC. OBC has less leucine and gamma‐aminobutyric acid (GABA) than OH. Leucine administration reverses metabolic disorders, improves glucose tolerance, and reduces hepatic steatosis and inflammation in adipose tissue (Macotela et al., 2011). In alcoholic liver disease (ALD), brain GABA levels are reduced, and more severe ALD is associated with lower cortical levels of GABA (Morley et al., 2020). These may explain the lower hepatoprotective effect of OBC compared to OH. Other ingredients besides these, particularly minerals, may also contribute to the hepatoprotective effect of OBC. Zinc, one of the most essential micronutrients associated with numerous biological functions, is abundant in oysters (Venugopal & Gopakumar, 2017). Zinc administration reduces ROS generation and EtOH‐induced CYP2E1 activity, and increases ADH activity and glutathione reductase activity in the liver (Mohammad et al., 2012). OBC appears to contain many components other than these that have beneficial effects on EtOH detoxication.

In our preliminary study, we analyzed the peptides present in OBC. Unfortunately, only carnosine (β‐alanyl‐L‐histidine) was discovered when the presence of the peptides was confirmed. Carnosine is a natural dipeptide that is abundantly distributed in the muscle and brain, and it has a powerful antioxidant effect. In the livers of binge EtOH‐administered rats, carnosine pretreatment reduces elevations in ALT and AST, as well as lipid peroxide (Artun et al., 2010). Carnosine also reduces oxidative stress and inflammation in liver injured rats (Alsheblak et al., 2016; Kalaz et al., 2014). Carnosine, on the other hand, is not a substantial component of OBC. OBC contains a variety of amino acids, among which it is rich in taurine. Several components found in OBC are predicted to work together to provide hepatoprotection.

Based on published papers (Goc et al., 2019; Iida & Hikichi, 1976), a taurine concentration of 45 mg/kg was used in this study. This concentration is similar to the concentration of taurine in OBC (37.54 mg/kg) preadministered to mice. It is difficult to accurately compare the concentration of substances between natural products because the concentration of components in the extract may vary depending on the manufacturing method and duration. However, these disadvantages can be overcome when a standard substance or main component of a natural product is found through component analysis and function test. In addition, if the mechanism of action of substances is analyzed together, it will be possible to further increase the utilization of natural products. In fact, since food intake may be the easiest way to maintain health and prevent disease, research is still needed to develop functional foods and analyze their value as pharmaceuticals. OBC has the potential to be used as a functional food that can prevent liver damage due to EtOH because the main ingredient and its mechanism are known in this study.

## 5 CONCLUSIONS

The preadministration of OBC enhanced EtOH metabolism and reduced ER stress, apoptotic signals, and inflammatory mediators in a single‐EtOH‐binge mouse model. Taurine administration showed similar effects to OBC. These results show that OBC protected against acute EtOH‐induced liver damage through the action of taurine. Our findings suggest that OBC could be an economically valuable substance and a functional food with hepatoprotective effects.

## CONFLICT OF INTEREST

The authors declare no conflicts of interest. The funding sponsors had no role in the design of the study; in the collection, analyses, or interpretation of the data; in the writing of the manuscript; or in the decision to publish the results.

## Supporting information

Supplementary Material1

Supplementary Material2

## Data Availability

The data that supports the findings of this study are available in the supplementary material of this article.
