# Supplementary material for: Oyster broth concentrate and its major component taurine alleviate acute alcohol‐induced liver damage
Source: Food Sci Nutr. 2022 Mar 29;10(7):2390–9. doi: 10.1002/fsn3.2847 (PMC9281932; doi:10.1002/fsn3.2847)
Supplement: Supplementary file 1 — Supplementary Material1 [file FSN3-10-2390-s001.pdf]

Supplementary Figure 1

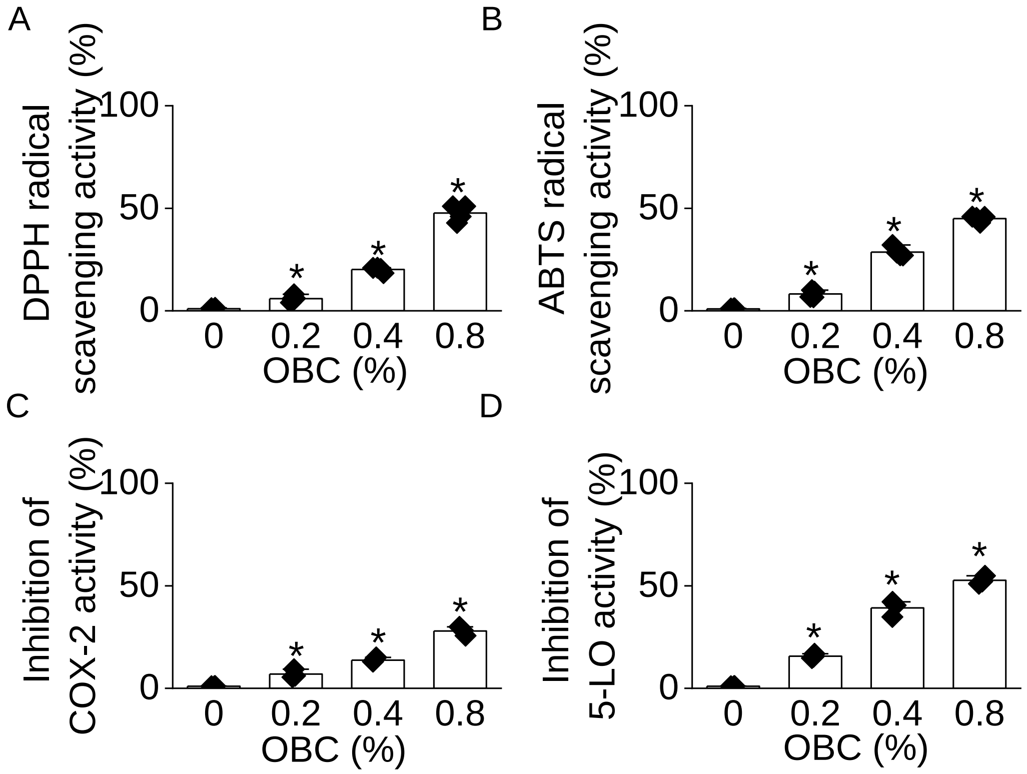


**Supplementary Figure 1. Antioxidant and anti-inflammatory activity of oyster broth concentrate (OBC).** (A and B) 2.2-diphenyl-1-picrylhydrazyl (DPPH) and 2,2-azinobis-(3-ethylbenzothiazoline-6-sulfonate) (ABTS) radical-scavenging activities of OBC. (C and D) Inhibition of cyclooxygenase-2 (COX-2) and 5-lipoxygenase (5-LO) activities by OBC. The activities of OBC were calculated by measuring absorbance. Each bar represents means ± SD of five independent experiments. *$p < 0.05$ compared to 0% of OBC (no treatment).

**Supplementary Table 1. Free amino acid content of oyster broth concentrate (OBC) and oyster hydrolysate (OH)**

| Amino acids | OBC (mM) | OH (mM) |
|---|---|---|
| **Taurine** | **1,499.6** | **49.0** |
| Glycine | 817.3 | 33.5 |
| Ammonia | 368.6 | 68.5 |
| Alanine | 359.6 | 73.4 |
| Proline | 282.3 | 15.7 |
| Glutamic acid | 182.6 | 7.4 |
| Arginine | 154.9 | 8.6 |
| Serine | 87.5 | 5.9 |
| Aspartic acid | 74.6 | 9.9 |
| β-Alanine | 70.2 | 10.5 |
| Leucine | 48.8 | 78.3 |
| Threonine | 43.4 | 26.7 |
| Asparagine | 41.6 | 15.2 |
| Phosphoserine | 33.3 | 1.4 |
| Valine | 32.0 | 41.9 |
| Lysine | 29.8 | 20.4 |
| Isoleucine | 26.8 | 34.3 |
| Tyrosine | 25.4 | 16.1 |
| Phenylalanine | 19.2 | 30.7 |
| Methionine | 19.2 | 20.1 |
| Histidine | 18.5 | 2.1 |
| Sarcosine | 1.9 | - |
| Ornithine | 1.8 | 11.7 |
| Ethanolamine | 1.7 | 2.6 |
| α-aminoisobutyric acid | 1.2 | 1.2 |
| GABA | 0.9 | 42.9 |
| α-aminobutyric acid | 0.7 | 0.9 |
| Tryptophan | 0.6 | 4.7 |
| Cystine | 0.3 | 0.2 |
| Hydroxylysine | 0.1 | 5.8 |
| Cystathionine | 0.1 | 1.5 |
| 1-methyhistidine | - | 9.8 |
| Citrulline | - | 4.3 |
| Homocysteine | - | 0.6 |
| Hydroxyproline | - | 34.6 |
| Urea | - | 86.6 |
| Total | 4244.5 | 777.0 |

The free amino acids were analyzed using an amino acid analyzer (Biochrom 20, LKB Biochrom Ltd., Cambridge, UK).
