# Supplementary material for: Oyster broth concentrate and its major component taurine alleviate acute alcohol‐induced liver damage
Source: Food Sci Nutr. 2022 Mar 29;10(7):2390–9. doi: 10.1002/fsn3.2847 (PMC9281932; doi:10.1002/fsn3.2847)
Supplement: Supplementary file 2 — Supplementary Material2 [file FSN3-10-2390-s002.docx]

**Supplementary Table 2. Values of EtOH-metabolic parameters for group administered taurine alone**

| Parameters | Values | |
| --- | --- | --- |
|  | Vehicle | Taurine |
| Latency to fall (sec)  ADH activity (mU/mL)  Acetaldehyde concentration (mM)  ALDH activity (mU/mL)  ALT (U/L)  AST (U/L)  Catalase level (U/mg)  CYP2E1 activity (pmol/mg/min)  ROS level (RFU)  Calcium concentration (mM) | 183.33 ± 4.73  3.44 ± 0.33  0.02 ± 0.03  36.46 ± 3.78  30.00 ± 3.00  149.00 ± 9.00  19.63 ± 1.17  276.95 ± 25.32  9035.40 ± 656.96  0.62 ± 0.13 | 183.00 ± 4.44  3.72 ± 0.14  0.02 ± 0.03  37.56 ± 4.63  29.00 ± 1.73  150.00 ± 7.21  19.40 ± 1.37  284.33 ± 21.03  9018.20 ± 572.68  0.65 ± 0.14 |

Data represents means ± SD of three independent experiments. Each group: n = 5-10. Latency to fall was measured by Rotarod performance test. ADH, alcohol dehydrogenase; ALDH, aldehyde dehydrogenase; ALT, alanine aminotransferase; AST, aspartate aminotransferase; CYP2E1, cytochrome P450 2E1; ROS, reactive oxygen species
